# The oncolytic adenovirus VCN-01 promotes anti-tumor effect in primitive neuroectodermal tumor models

**DOI:** 10.1038/s41598-019-51014-1

**Published:** 2019-10-07

**Authors:** Marc Garcia-Moure, Naiara Martinez-Velez, Marisol Gonzalez-Huarriz, Lucía Marrodán, Manel Cascallo, Ramón Alemany, Ana Patiño-García, Marta M. Alonso

**Affiliations:** 1 Navarra’s Health Research Institute (IDISNA), Pamplona, Spain; 2 Program in Solid Tumors and Biomarkers, Foundation for the Applied Medical Research, Pamplona, Spain; 3 Department of Pediatrics, University Hospital of Navarra, Pamplona, 31008 Spain; 4 VCN Biosciences, Sant Cugat del Vallés, 08174 Barcelona, Spain; 5 Translational Research Laboratory, IDIBELL-Institut Catalá d’Oncologia, L’Hospitalet de Llobregat, 08907 Barcelona, Spain

**Keywords:** CNS cancer, Cell death

## Abstract

Last advances in the treatment of pediatric tumors has led to an increase of survival rates of children affected by primitive neuroectodermal tumors, however, still a significant amount of the patients do not overcome the disease. In addition, the survivors might suffer from severe side effects caused by the current standard treatments. Oncolytic virotherapy has emerged in the last years as a promising alternative for the treatment of solid tumors. In this work, we study the anti-tumor effect mediated by the oncolytic adenovirus VCN-01 in CNS-PNET models. VCN-01 is able to infect and replicate in PNET cell cultures, leading to a cytotoxicity and immunogenic cell death. *In vivo*, VCN-01 increased significantly the median survival of mice and led to long-term survivors in two orthotopic models of PNETs. In summary, these results underscore the therapeutic effect of VCN-01 for rare pediatric cancers such as PNETs, and warrants further exploration on the use of this virus to treat them.

## Introduction

Brain tumors are the leading cause of death in children from Western countries, as they are responsible for an important fraction of the natural occurring mortality in children and adolescents^1^. Primitive neuroectodermal tumors from central nervous system (CNS-PNETs or PNETs) are rare pediatric brain malignancies affecting children^1,2^.

CNS-PNET is a heterogeneous group of brain embryonal tumors which encompasses all CNS embryonal malignancies that cannot be diagnosed as medulloblastoma, atypical teratoid/rhabdoid tumor (AT/RT) or embryonal tumors with multilayered rosettes (ETMRs)^3^. Histologically, CNS-PNET are characterized by the presence of small poorly differentiated cells as well as a mixed population of both glial and neuronal lineages^2,4^. Despite that CNS-PNETs represent only the 2% of brain tumors in patients under 14 years old, they cause up to 7.5% of deaths related to primary brain tumors in this population^1^, thus underscoring the aggressiveness of these tumors. For the most, PNETs are treated with high-dose chemotherapeutic schedules according to the current high-risk medulloblastoma protocols^5,6^, leading to the development of severe side effects. Therefore, it is clear the need to find new strategies for the treatment of PNETs.

Oncolytic adenoviruses have emerged in the last years as an innovative tool for the treatment of solid tumors, as they selectively replicate in and kill tumor cells. As the oncolytic adenovirus replicates, the viral progeny infects nearby tumor cells and engages a new lytic cycle that leads to partial or total reduction of the tumor mass. However, the presence of a dense extracellular matrix (ECM) in the tumor is a physical barrier that hinders the viral spread. To overcome this problem, some adenoviruses have been modified to express matrix-degradation enzymes and facilitate viral dissemination^7,8^. VCN-01 oncolytic virus is a derivative from the cancer-selective adenovirus ICOVIR-15K, which has been modified to achieve tumor specificity^9^. In regard to infectivity, the addition of an integrin-binding motif RGD in the fiber shaft facilitates the attachment of the virus to cells through α_v_β_3/5_ integrins, resulting in an improved infection of cancer cells, which often downregulate CAR, the natural adenoviral receptor^10^. VCN-01 holds also modifications in the immediate early E1A viral gene to limit its replication to tumor cells. First, E1A has a 24-bp deletion in its pRb binding site; therefore, the lytic viral cycle can be triggered only in pRb-deficient cells. In addition, E1A promoter holds eight E2F-1 responsive elements that stablish a positive feedback in cancer cells which results in a boosted replication of the virus^11^.

In addition, VCN-01 carries a secreted form of the human PH20 hyalorunidase gene, in order to degrade hyaluronic acid (HA) in the ECM and facilitate the viral particles to spread within the tumor. The effectiveness of this virus has already been proven in preclinical models of pancreatic adenocarcinoma^9^, osteosarcoma^12^, high grade glioma^13^, and retinoblastoma^14^, demonstrating a reduction in tumor growth and extended overall survival in VCN-01 treated mice with no signs of toxicity observed. According to these data, particularly those obtained in high-grade glioma, we hypothesized that VCN-01 may be also a good candidate for virotherapy in the treatment of PNETs.

Here, we describe the anti-tumor effect mediated by the oncolytic adenovirus VCN-01 in two CNS-PNET models. *In vitro*, VCN-01 infected and replicated in PNET stable cell lines efficiently, eventually leading to cytolysis of the tumor cells. Additionally, mice bearing supratentorial CNS-PNET xenografts showed an increase in overall survival upon intratumoral administration of VCN-01. Altogether, these results uncover VCN-01 as a new promising alternative for the treatment of these aggressive malignancies.

## Materials and Methods

### Cell lines and culture conditions

PFSK-1 (ATCC, Manassas, VA, USA; CRL-2060^TM^) was maintained in RPMI-1640 (Gibco, Life Technologies, NY, USA). SK-PN-DW (ATCC, CRL-2139^TM^), HEK293 (ATCC, CRL-1573^TM^) and A549 (ATCC, CRL-185^TM^) DMEM (Gibco, Life Technologies, NY, USA). All culture media were supplemented with 10% fetal bovine serum, and cultures were incubated in a humidified atmosphere containing 5% CO_2_. All cell lines were routinely tested for mycoplasma (Mycoalert mycoplasma detection kit; Lonza) and authenticated at the CIMA Genomic Core Facility (Pamplona, Spain) using DNA profiling.

### Viruses

VCN-01 was propagated in A549 cultures and purified by double ultracentrifugation in CsCl gradient. AdTLRGDK was kindly provided by Dr Ramon Alemany.

### Flow cytometry

PNET cell lines PFSK-1 (ATCC, Manassas, VA, USA; CRL-2060^TM^) and SK-PN-DW (ATCC, CRL-2139^TM^) were stained with unlabeled monoclonal antibodies recognizing the adenoviral receptors CAR (Merck Millipore, Temecula, CA, USA; 05–644) α_v_β_3_ integrin (Merck Millipore; CBL544) and α_v_β_5_ integrin (R&D Systems Inc., Minneapolis, MN, USA; MAB2528), and donkey anti-mouse IgG1-AlexaFluor488 (Thermo Fisher Scientific, Waltham, MA, USA; A-21202) as secondary antibody. The samples were next analyzed using the FACSCanto^TM^ II system (BD Biosciences, Franklin Lakes, NJ, USA) and FACSDiva software (BD Biosciences).

### Infectivity assays

PFSK-1 or SK-PN-DW were plated at 2 × 10^5^ cells per well, and infected with AdTLRGDK (kindly provided by Dr Ramon Alemany) at MOIs of 0, 0.1, 1, 10 or 100 PFU/cell. 24 and 48 h later, the cultures were harvested and expression of GFP was measured by flow cytometry.

### Viral replication assays

PFSK-1 and SK-PN-DW cells were seeded at a density of 10^5^ cells per well in 6-well plates, and subsequently infected at MOIs of 1 or 10 PFU/cell. 72 hours post-infection cells and medium were harvested and freeze–thawed three times, and PFUs were titrated in HEK293 by the anti-hexon staining-based method^15^.

### Cell viability assay

PFSK-1 or SK-PN-DW were plated in 96-well plates at a density of 2 × 10^3^ cells per well, and then infected with VCN-01 at different MOIs ranging from 0 to 40. Cell viability was assessed five days later using CellTiter 96® Aqueous One Solution Cell Proliferation Assay (Promega, Fitchburg, WI, USA; G3581) as previously described^16^. Dose–response curves were analyzed using GraphPad Prism 8 (Statistical Software for Sciences) to determine the IC_50_ of VCN-01 in these cells. The IC_50_ value is the median-effect dose (dose affecting 50% of the cells, i.e., 50% survival).

### Propidium iodide staining

PNET cell lines were plated in 6-well plates at 2 × 10^5^ cells per well, and then infected with VCN-01 at the same doses as above. Five days after the infection, cells were harvested and stained using propidium iodide containing NucleoCassettes™ (ChemoMetec, Allerød, Denmark; 941–0002). Percentage of alive cells were determined with the NucleoCounter® (ChemoMetec).

### Damage Associated Molecular Pattern (DAMPs) secretion

PFSK-1 or SK-PN-DW cell (2 × 10^5^ per well) were infected with VCN-01 at 3.5 (PFSK-1) or 7.0 (SK-PN-DW) MOIs. Three days later, the concentration of Hsp90α (Enzo Life Sciences Inc; ADI-EKS-895) and HMGB1 (IBL International, Hamburg, Germany; ST51011) were measured by ELISA. ATP release was quantified by luminometry using the ENLITEN® ATP Assay System (Promega; FF2000). Calreticulin translocation to cell membrane was determined by immunofluorescence. Briefly, 2 × 10^4^ PFSK-1 or SK-PN-DW cells were seeded in glass coverslips (previously coated with laminin for SK-PN-DW). Next day, cultures were infected with VCN-01 at their respective MOIs (or mock infected for negative control), and 4 hours after the infection cells were fixed in 50% ethanol, 2% PEG 6000. Immunostaining was performed using anti-calreticulin primary antibody (1:250; Abcam; ab2907) and anti-rabbit secondary antibody conjugated with AlexaFluor594 (1:500; Thermo Fisher Scientific, Walthan, MA, USA; A-21207).

### Immunoblotting

For the immunoblotting assays, the samples were subjected to SDS-Tris-glycine gel electrophoresis. The membranes were incubated with the following antibodies: E1A, (1:1000; Santa Cruz Biotechnology, Santa Cruz, CA, USA; sc-430), fiber (1:1000; Thermo Fisher Scientific, Walthan, MA, USA; MS-1027), and GRB-2 (1:1000; BD Biosciences, Franklin Lakes, NJ, USA; 610111). The membranes were developed according to Amersham’s enhanced chemiluminescence protocol.

### Animal studies

5 × 10^5^ PFSK-1 or SK-PN-DW cells were engrafted into the caudate nucleus of 4–6 weeks female athymic nude (nu/nu) mice (Envigo, Huntingdon, UK) following the guide-screw system described by Lal *et al*.^17^. Briefly, a hollowed bolt was implanted 2.5 mm lateral and 1 mm cranial from Bregma. Tumor cells were then injected through the hollow at 2 mm depth (3.5 mm including bolt height). On the third day after implantation of cells, animals were randomized and treated with 5 μL of either PBS or VCN-01 (10^8^ PFU/animal) through the same bolt at 2 mm depth. Animals were sacrificed when symptomatology of the disease was evident (loss of weight, hunched position), and survival curves were plotted according to the Kaplan-Meier method, using the log-rank test to compare median survival. Ethical approval for the animal studies was granted by the Animal Ethical Committee of the University of Navarra (CEEA) under the protocol number CEEA/094-15. All animal studies were performed at the veterinary facilities of the Center for Applied Medical Research in accordance with institutional, regional, and national laws and ethical guidelines for experimental animal care.

### Immunohistochemical analysis

Paraffin-embedded sections of mice brains (4 µm thickness) were were stained by hematoxylin/eosin method, or immunostained with anti-hexon antibody (1:2000; Merck Millipore; AB1056), biotinylated hyaluronic acid binding protein (1:400; Merck Millipore; 385911), anti-F4/80 antibody (1:400; Cell Signaling; 70076) and Iba1 (1:4000; Wako; 019-19741). For the immunohistochemical staining, Vectastain ABC kits (Vector Laboratories Inc., Burlingame, CA) were used according to the manufacturer’s instructions. Hyaluronic acid, F4/80 and Iba1 stained area was quantified using the Fiji platform^18^ in three different regions of the samples.

### Statistical analysis

For the *in vitro* experiments, the data are expressed as the mean ± SD, and the comparisons were performed using two-tailed Student *t*-tests. The survival in different treatment groups was compared using a log-rank test. The program GraphPad Prism 8 (Statistical Software for Sciences) was used for the statistical analyses.

## Results

### Expression of adenoviral receptors allows VCN-01 to infect and replicate in PNET cells, promoting cell death and release of immunogenic cell death markers

**Figure 1:**
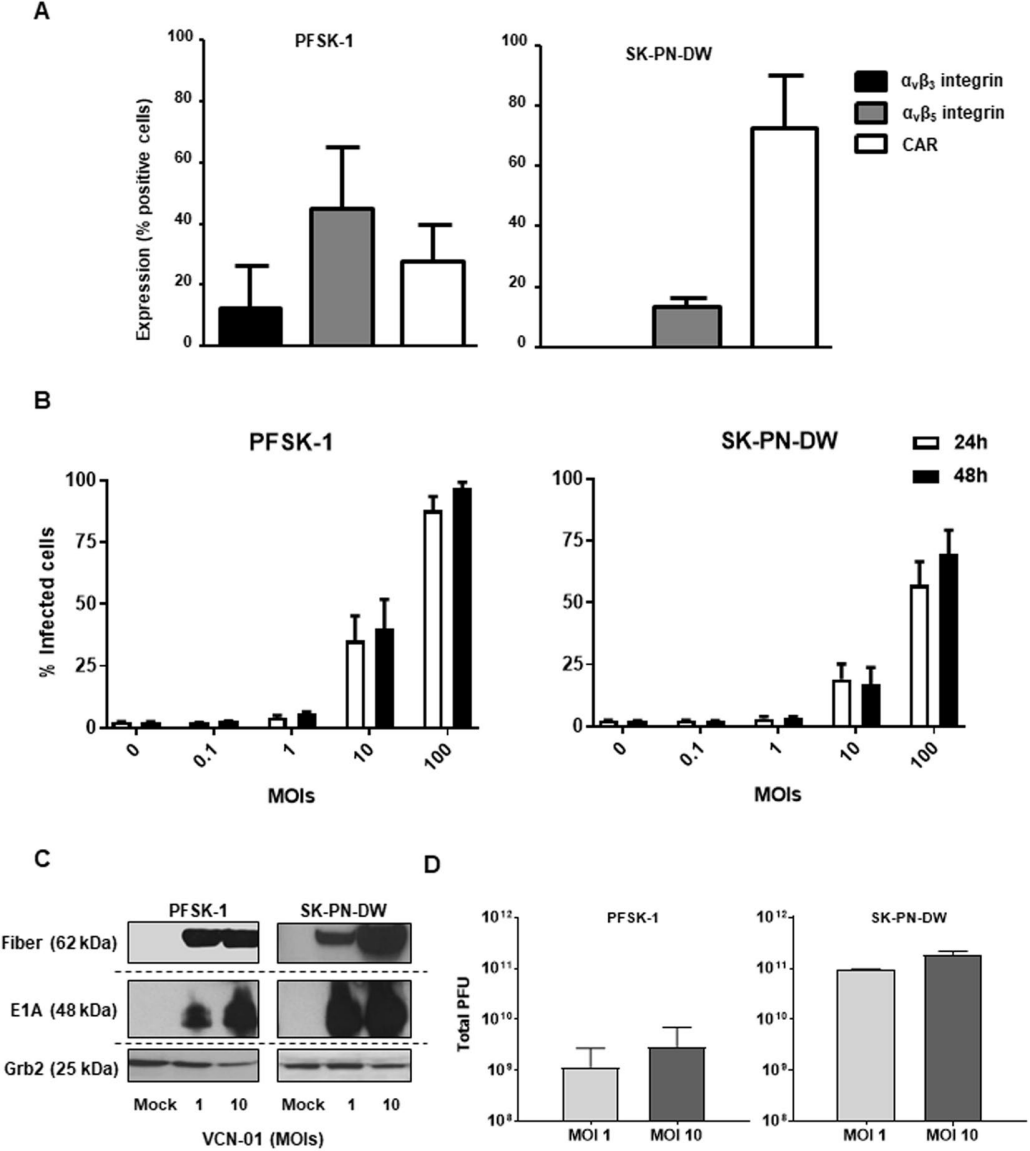
Characterization of VCN-01 in PNET cell lines *in vitro*. (**A**) Expression of adenoviral receptors CAR, α_v_β_3_ integrin and α_v_β_5_ integrin in CNS-PNET cell lines PFSK-1 (left) and SK-PN-DW (right). Graph shows the percentage of stained cells for each receptor (Mean ± SD; *n* = 3). (**B**) PFSK-1 and SK-PN-DW (200,000 cells) infected with the GFP expressing vector AdTLRGDK at MOIs of 0, 0.1, 1, 10 or 100 PFU/cell. Graph indicates the percentage of GFP positive cells expression measured by flow cytometry at 24 h (white bars) or 48 h (black bars) after the infection (Mean ± SD; *n* = 3). (**C**) Detection of the viral proteins E1A and fiber in whole-cell lysates 48 h after being from PFSK-1 and SK-PN-DW from VCN-01 infected PFSK-1 and SK-PN-DW cultures. Grb2 was used as loading control protein. Blots from different parts of the same gel have been grouped to improve clarity of the image. (**D**) Viral titers in PFSK-1 and SK-PN-DW (50,000 cells/well) cultures 72 h after being infected with VCN-01 at MOIs 1 and 10. Bars represent total PFUs contained in the lysates (Mean ± SD; *n* = 3).

VCN-01 is a derivative of serotype 5 human adenovirus, and its natural adenoviral primary receptor is the Coxsackie and Adenovirus Receptor (CAR)^19,20^, a cell to cell adhesion molecule which is usually downregulated in tumors^21,22^. VCN-01 has been modified to also bind α_v_β_3/5_ integrins by the addition of the integrin binding motif RGD in the fiber protein^9^, thus the virus is able to infect tumor cells through integrins regardless of their CAR status. We determined the presence of the adenovirus receptors CAR, α_v_β_3_, and α_v_β_5_ integrins in PNET cell lines PFSK-1 and SK-PN-DW by flow cytometry in order to ensure their susceptibility VCN-01 infection. Adenovirus receptors were expressed in both PNET models (Fig. 1A); however, the levels of the three receptors differed between the cell lines. In PFSK-1, the receptor CAR was detected in 27.6% of the cells, α_v_β_3_ integrin in 12.7% and α_v_β_5_ integrin in 44.9% of the cells. On the other hand, CAR expression was detected in most of SK-PN-DW cells (72.5%), although only 13.3% of the cells were positive for α_v_β_5_ integrin. Integrin α_v_β_3_ was below detection limit in SK-PN-DW. According to these results, PNET cell lines are positive for adenoviral receptors, thus being permissible to VCN-01 infection. In order to confirm the infectivity of VCN-01 in PNET cell lines, PFSK-1 and SK-PN-DW cultures were infected at increasing MOIs with the virus AdTLRGDK, a non-replicative adenovirus containing the same capsid modifications present in VCN-01^23^. As AdTLRGDK encodes a green fluorescent reporter, the percentage of infected cells was measured 24 h later by flow cytometry. PFSK-1 and SK-PN-DW cultures were infected by VCN-01, being PFSK-1 more efficiently transduced by the virus (Fig. 1B). As this is a non-replicative virus, fluorescence was measured again at 48 h post-infection in order to allow infected cells to express more GFP, thus increasing the sensitivity of the experiment (specially in low MOI conditions). No remarkable differences were observed between 24 and 48 h. The expression of CAR and integrins allows VCN-01 to infect both PNET cell lines efficiently, being PFSK-1 more sensitive to VCN-01 infection. This difference in infectivity between both cell lines is in agreement with a higher expression of α_v_β_3/5_ integrins in PFSK-1 as integrins, but not CAR, are required for the internalization of the adenovirus^24^. However, we cannot conclude the existence of a direct link between infectivity and integrin expression only with this result.

VCN-01 derives from the conditionally replicative adenovirus ICOVIR15K, whose replication requires an impaired pRb pathway^10^. In order to evaluate viral cycle progression in PNET cells, PFSK-1 and SK-PN-DW were infected with VCN-01 at MOIs 1 and 10 or mock infected. The presence of the viral proteins E1A (early protein) and fiber (late protein) was assessed by Western blot 48 hours after the infection. E1A is a viral early protein which is constitutively activated as soon as the virus reaches the cell nucleus^25^, and for this reason the presence of E1A protein indicates viral infection. On the other hand, the adenoviral fiber protein is a viral late protein, and its gene is under control of the Major Late Promoter, which is activated after the replication of the viral genome^26^. Thus, the detection of fiber protein is indicative of the progression of the viral cycle. Expression of both E1A and fiber was detected in infected PNET cell lines 72 h after infection with VCN-01 in a dose-dependent manner (Fig. 1C), suggesting the replication of the adenovirus in these cell lines. In virotherapy, the generation of infectious capsids is crucial because the viral progeny must be able to infect nearby uninfected tumor cells in order to start a new lytic cycle and maximize the tumor shrinkage. To demonstrate the production of fully mature viral progeny, infectious viral titers were determined in PFSK-1 and SK-PN-DW cultures 72 hours after being infected by VCN-01 at MOIs 1 and 10. Generation of VCN-01 infectious particles was observed in both cell lines (Fig. 1D). In PFSK-1, the titer of newly generated infectious virions reached 1.2 × 10^9^ and 2.9 × 10^9^ PFU at MOIs 1 and 10, respectively. Replication of VCN-01 in SK-PN-DW was much more efficient, since we obtained infectious titers of 9.4 × 10^10^ and 1.9 × 10^11^ PFU at MOIs 1 and 10, respectively. Nevertheless, all titers were above the input levels of virus (10^5^ and 10^6^ PFUs for MOIs 1 and 10, respectively), thus demonstrating a vigorous replication of VCN-01 in both PNET cell lines.

**Figure 2:**
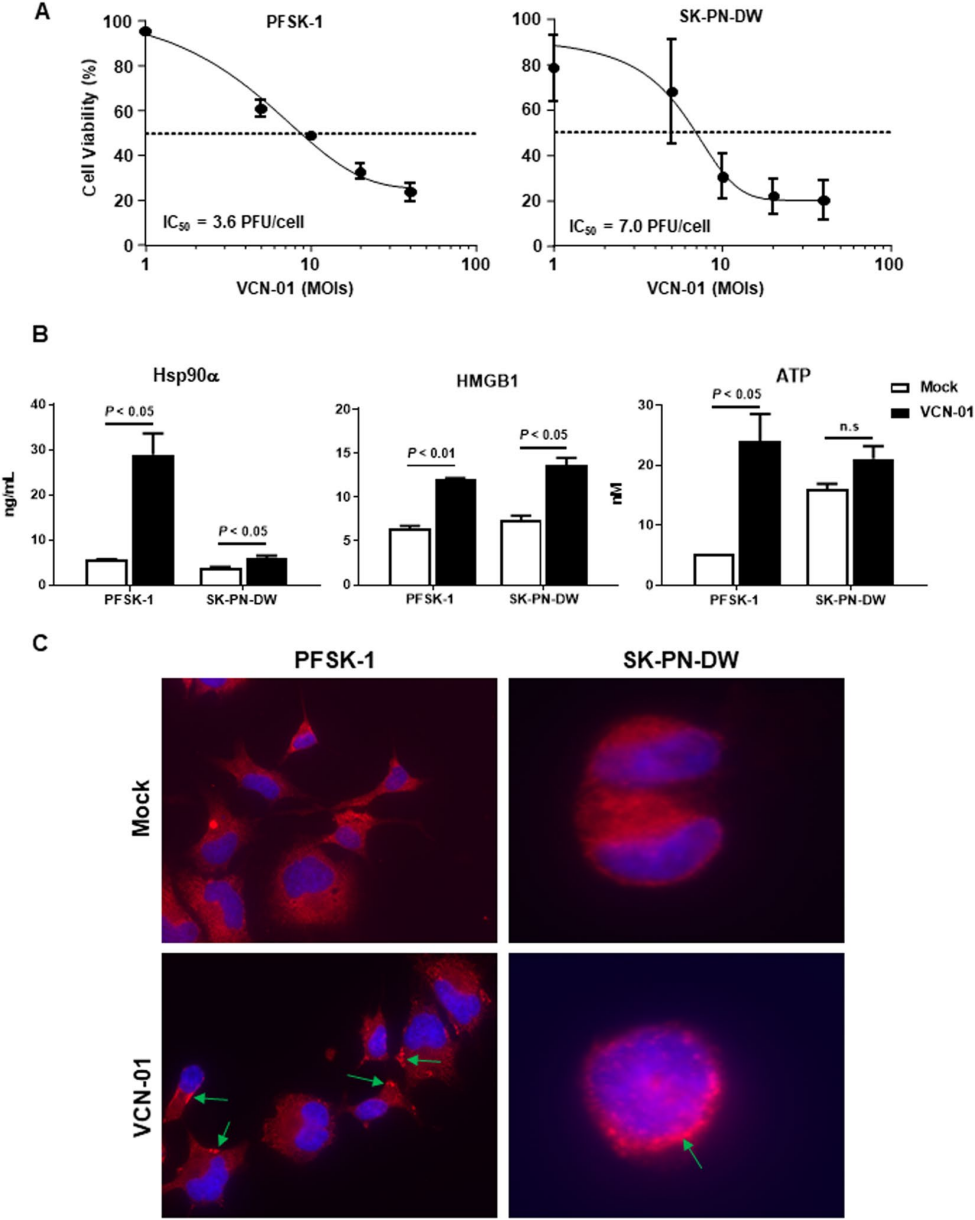
VCN-01 treatment results in cytotoxicity and immunogenic cell death. (**A**) Viability of PFSK-1 or SK-PN-DW cultures infected with VCN-01 at different MOIs ranging from 0 to 40 PFU/cell at 5 days post-infection. Graph indicates the percentage of viable cells relative to non-infected cultures, considered as 100% of viability (Mean ± SD; *n* = 3). (**B**) Measurement of DAMPs Hsp90α, HMGB1 and ATP in supernatants obtained from PFSK-1 and SK-PN-DW cultures three days after being infected with VCN-01 at their respective IC_50_ (Mean ± SD; *n* = 3). (**C**) Representative images of calreticulin staining (red signal) in PFSK-1 (left) and SK-PN-DW (right) 4 h after being infected with either VCN-01 (bottom) or mock infected (top). Nuclei were stained in blue (DAPI), and green arrows indicate the location of calreticulin clusters in the cell membrane.

Once determined an efficient replication of VCN-01 in PNET cells, we wondered at what extent this replication of the virus would lead to tumor cell death. For this purpose, PFSK-1 and SK-PN-DW cells were infected with VCN-01 at increasing doses from 0 to 40 MOIs and cell viability was measured five days after the infection to determine the IC_50_. Both PNET cell lines were susceptible to oncolysis mediated by the virus in a dose-dependent response (Fig. 2A). The IC_50_s MOIs estimated were 3.6 in PFSK-1 and 7.0 in SK-PN-DW being PFSK-1 slightly more sensitive to VCN-01 mediated cell death. The MTS assay we have used to evaluate viability is dependent on metabolic activity, which can be altered by the adenovirus. Therefore, we also used propidium iodide staining to confirm the lytic effect of VCN-01 (S1 Fig). The results obtained with this technique also demonstrate that VCN-01 is able to mediate cell death. However, we could not determine an IC_50_ value because even at the lowest MOI tested, the percentage of cells compared to non-infected cultures was below 50% (40% and 12% for PFSK-1 and SK-PN-DW, respectively). For this reason, IC_50_ obtained by MTS assay were used as a reference in further experiments.

In order to evaluate the ability of VCN-01 to induce immunogenic cell death (ICD) in PNET tumors, PFSK-1 and SK-PN-DW were infected at their respective 5-day IC_50_ dose level (Fig. 2A), and the concentration of the ICD markers; heat-shock protein 90 (Hsp90α), high-mobility group proteins B1 (HMGB1) and ATP was measured in the supernatants three days later. In both PNET models, the infection with VCN-01 mediates a significant increase in secreted Hsp90, HMGB1 and ATP, ranging from 1.6 to 5.2 times higher than in mock infected cultures (Fig. 2B). For SK-PN-DW cell line, although the differences in ATP content in VCN-01 treated vs mock infected (control) cultures are not statistically significant, we do observe a tendency in agreement with the rest of ICD markers. Translocation of calreticulin from endoplasmic reticulum to cell surface is another well know early hallmark that triggers ICD as well as phagocytosis of the damaged cell^27^. In order to evaluate the effect of VCN-01 on this hallmark, PNET cell lines were infected with VCN-01 at their respective IC_50_s and 4 h later the location of calreticulin was determined by immunofluorescence. We observe the presence of calreticulin clusters in the cell membrane surface of VCN-01 infected cells that were not noticeable in non-infected cultures (Fig. 2C). All these results together, suggest the trigger of ICD in PFSK-1 and SK-PN-DW cell lines after infection with VCN-01.

### VCN-01 promotes an extended overall survival in mouse bearing orthotopic PNET

**Figure 3:**
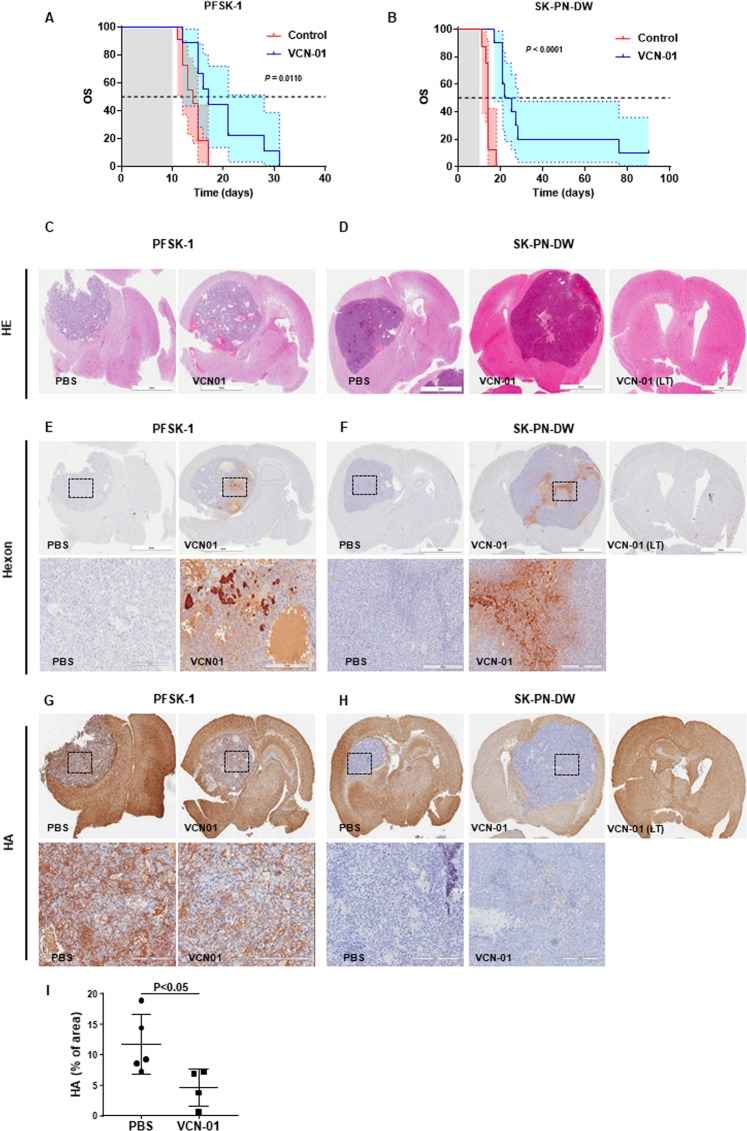
Anti-tumor effect of VCN-01 in PNET xenograft models. (**A,B**) Survival curves of mice bearing orthotopic PFSK-1 cells (A) or SK-PN-DW (**B**) tumors. Graphs represent overall survival (OS) with 95% confidence interval (blue and red shades) of mice treated with VCN-01 (blue line) or mock treated (red line). (**C**–**G**) Brain sections from mice bearing xenografts from either PFSK-1 (**C**) or SK-PN-DW (**D**) cells. Slices were stained with hematoxylin/eosin staining, or immunostained with anti-hexon (E, F) and hyaluronic acid binding protein. (**G**,**H**) Tumor regions (dotted square) have been magnified 10X in bottom pictures. VCN-1 (LT) (right column) corresponds to a long-term survivor. (**I**) Quantification of HA stained area (relative to total tumor area) in brain sections from mice bearing PFSK-1 tumors treated with either PBS or VCN-01 (Mean ± SD; *n* = 4/5).

Our next aim was to evaluate whether VCN-01 *in vitro* antitumor would also lead to an *in vivo* effect in two orthotopic xenograft murine models. Mice bearing supratentorial (cerebrum) PFSK-1 or SK-PN-DW tumors received a single administration of VCN-01 (10^8^ PFU/mouse) or PBS *in situ* three days after implantation of the cells. Median survival obtained for control (PBS) treated mice was 14 days in both PNET models (Fig. 3A,B). Nevertheless, VCN-01 treated mice median survival was extended up to 17 days in PFSK-1 model (*P* = 0.011) and 23.5 days in SK-PN-DW model (*P* < 0.0001). Moreover, one mouse survived until the end of the experiment (90 days) without any evidence of tumor growth. However, due to the lack of tumor imaging in this experiment, we must be cautious about this long-term survivor, as we have no evidence of tumor engraftment in this mouse.

Hematoxylin/eosin staining showed the presence of large tumor masses in brains obtained from mice bearing PFSK-1 (Fig. 3C) or SK-PN-DW tumors (Fig. 3D). In VCN-01 treated brains, immunohistochemical hexon staining (Fig. 3E,F) revealed areas of active viral replication within the tumor. Of note, these replicating areas overlap with highly necrotic regions, which is indicative of the oncolytic activity mediated by the virus. Neither tumor nor adenoviral replication were detected in SK-PN-DW long-term survivor.

VCN-01 encodes the secreted PH20 human hyaluronidase, which degrades hyaluronic acid (HA) present in the extracellular matrix and facilitates the spread of viral particles through the tumor mass. Brain sections from the VCN-01 and PBS treated animals were stained for HA with the aim to evaluate the effect of the virus in degrading extracellular matrix. In PFSK-1 tumors, we detect a remarkable expression of HA (Fig. 3G) spanning about 12% of the tumor area in PBS treated mice. However, in VCN-01 treated mice, the area occupied by HA drops significantly to 4.6% of the tumor, thus indicating that VCN-01 actually is mediating HA degradation (Fig. 3G). On the other hand, SK-PN-DW tumors were negative for HA in their extracellular matrix (Fig. 3H) and therefore we could not evaluate the effect of secreted PH20 hyaluronidase in this model. Therefore, the antitumor effect observed in this cell line is related to the oncolytic effect of the virus.

**Figure 4:**
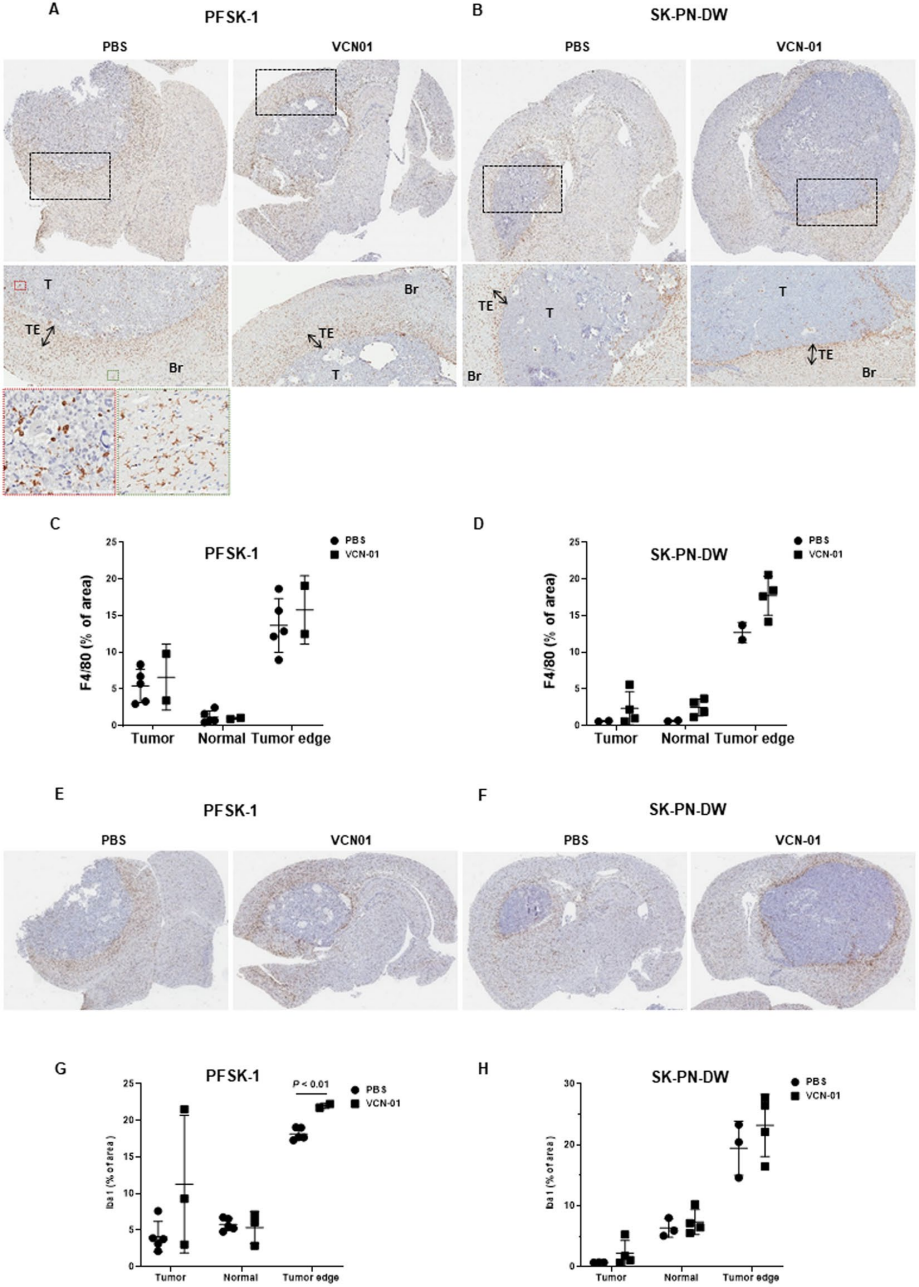
VCN-01 stimulate macrophage recruitment to the tumor periphery. (**A,B**) Brain sections from PBS and VCN-01 treated mice bearing xenografts from either PFSK-1 (**A**) or SK-PN-DW (**B**) cells immunostained with anti-F4/80 antibody. Tumor regions (dotted black squares) have been magnified in the middle panel, and labels T, TE and Br indicate tumor stroma, tumor edge and normal brain parenchyma, respectively. Red and green dotted squares have been amplified in the bottom panel to illustrate examples of amoeboid and ramified macrophages, respectively. (**C,D**) Quantification of F4/80 stained area (relative to total tumor area) in brain sections from mice bearing PFSK-1 (**C**) or SK-PN-DW (**D**) tumors treated with either PBS or VCN-01 (Mean ± SD). (**E–F**) Brain sections from PBS and VCN-01 treated mice bearing xenografts from either PFSK-1 (**E**) or SK-PN-DW (**F**) cells immunostained with anti-Iba1 antibody. (**G,H**) Quantification of Iba1 stained area (relative to total tumor area) in brain sections from mice bearing PFSK-1 (**G**) or SK-PN-DW (**H**) tumors treated with either PBS or VCN-01 (Mean ± SD).

This *in vivo* study was performed in immunodeficient mice to allow engraftment of our human PNET models. Yet, athymic nude mice still produce immune cells from the myeloid lineage, and we wondered whether VCN-01 treatment would affect the recruitment and infiltration of macrophages/microglia in the tumor. We observed that brain sections from VCN-01 or PBS treated mice were infiltrated by macrophages by expression of F4/80 positive cells (PFSK-1; Fig. 4A and SK-PN-DW; Fig. 4B). A closer look to images reveals that F4/80 positive cells also accumulate in the tumor edge, spanning a 200–300 µm wide region surrounding the tumor (Fig. 4A,B, middle panel). Interestingly, tumor stroma was enriched in amoeboid shaped F4/80 cells (reactive phenotype) in comparison to normal brain parenchyma, where most of F4/80 cells show a ramified morphology corresponding to resting microglia (Fig. 4A, bottom panel). In the tumor edge, we found a mixed population of reactive and resting F4/80 positive cells, suggesting a transitional zone. Quantification of F4/80 stained area showed no statistically significant differences between PBS and VCN-01 treated mice (Fig. 4C,D), although we observed a tendency to a higher degree of infiltration in the VCN-01 group, especially in the tumor edge. To further validate these data we assessed the expression of another macrophage/microglia marker, Iba1 (Fig. 4E–H). We found an accumulation of macrophages/microglia in the tumor periphery, and this infiltration was significantly enhanced by VCN-01 treatment in PFSK-1 tumors (*P* < 0.01). We observed a similar tendency in the tumor edge of SK-PN-DW, but, again, not statistically significant.

Altogether, these results allow us to postulate VCN-01 as an alternative approach for the treatment of CNS-PNETs.

## Discussion

CNS-PNETs are highly aggressive pediatric brain tumors that, to date, are being treated using the chemo and radiotherapeutic protocols established for high-risk medulloblastoma, thus causing severe side effects to these patients. In this preclinical study, we use two different CNS-PNET models to evaluate the feasibility of using the oncolytic adenovirus VCN-01 as a therapeutic alternative for this disease.

*In vitro*, we confirm the sensitivity of PNET cell cultures to VCN-01 infection and replication. Both models express the receptors required to adenovirus attachment and infection of the target cell. In regards to the pRb pathway, a study published by Li M *et al*. shows that about 25% of CNS-PNETs tumors analyzed contain focal amplifications in CDK/CYCLIND genes^28^. Although we have not evaluated the status of this pathway, the adenoviral replication in PFSK-1 and SK-PN-DW cell lines are indicative of an impaired Rb pathway in both cell lines or expression of E2F-1. E2F-1 is a transcription factor E2F-1 that drives the replication of VCN-01 and that is free in replicating cells. In addition, it must be considered that although CNS-PNET was formerly considered as a single disease, molecular profiling of these tumors has showed up that CNS-PNET tumors are a heterogeneous group of malignancies^3,29^. Because major changes in the classification of tumors as CNS-PNET are still ongoing, it is challenging to determine yet which tumors are actually CNS-PNETs and, therefore, what is the real extent of pRb pathway alterations in CNS-PNETs.

Infection of VCN-01 in PNET cultures promotes tumor cell death in a dose-dependent manner, which is related to expression of viral proteins and replication. We have observed also the secretion of ICD markers Hsp90α, HMGB1 and ATP, as well as translocation of calreticulin to cell membrane. ICD is a particular mechanism of cell death that mediates the stimulation of the adaptive immune system through exposure of DAMPs by the dying cell under some cell-damaging agents such as chemotherapy or viral infections^30^. In a recent clinical trial using the oncolytic adenovirus Delta-24-RGD for the treatment of recurrent glioma it was clear that the anti-tumor effect mediated by the virus lies not only in the replication of the virus, but also in the awakening of the immune response against the tumor^31,32^. However, although we have detected secretion of DAMPs in VCN-01 infected PNET cells, whether these signals will eventually lead to a real ICD or not is something that should be further explored in immunocompetent models. Unfortunately, the paucity of viable PNET murine models is a major drawback and difficults the study of the immune response against PNET tumors, as the engraftment of human PNET can only be achieved in immunodeficient mice.

In our preclinical study, we have evaluated the anti-tumor effect of VCN-01 after direct intratumoral injection of the virus in orthotopic xenograft murine models using both PFSK-1 and SK-PN-DW CNS-PNET models. Despite the aggressiveness of these models (median survival of 14 days in mock treated animals), the administration of VCN-01 demonstrates its therapeutic benefit with a single injection of the virus. Surprisingly, we have found major differences in hyaluronic acid content in PFSK-1 and SK-PN-DW tumors. To our knowledge, there is no available data about the expression of HA in patient tumor samples; therefore, more studies should be carried out to evaluate the therapeutic benefit of using an oncolytic virus encoding the PH20 versus non-armed oncolytic viruses. In regard to brain tumors, our group have previously proved the efficacy of VCN-01 in high-grade glioma models^13^. Although VCN-01 has been tested in different studies, including an ongoing clinical trial for pediatric patients with refractory retinoblastoma (NCT03284268), to our knowledge this is the first preclinical evaluation of VCN-01 in pediatric brain tumors.

Both PNET models are positive for macrophages/microglia infiltration. The markers used in this report (F4/80 and Iba1) do not allow us to distinguish between resident microglia and recruited peripheral macrophages. However, based in their morphology we can identify resting and reactive cells according to their ramified (mostly in brain parenchyma) and amoeboid shape (mostly in the tumor stroma and tumor edge),. Interestingly, the highest density of macrophages was found in the periphery of the tumors rather than infiltrated in the tumor stroma. No clear differences were observed in tumor infiltration of macrophages/microglia between VCN-01 and PBS treated mice. It must be taken into account that brain samples were collected at different days when symptomatology of the disease appeared rather than at a specific time. Nevertheless, with both markers we have observed that VCN-01 administration increases monocyte recruitment to the tumor edge. Cautiously and taking our results as starting point, we hypothesize that macrophages actually respond and migrate to the tumor, but once there they find such an immunosuppressive microenvironment in the tumor stroma that their infiltration is hampered, even in the presence of an immunostimulating agent as the oncolytic adenovirus VCN-01. This hypothesis paves the way to future studies to evaluate the therapeutic benefit of VCN-01 in combination with immune-checkpoint blocking agents that may help to overcome the inhibition of macrophages recruited by the virus.

In summary, we demonstrate in this preclinical study that VCN-01 oncolytic adenovirus could be a feasible therapeutic choice for the treatment of PNETs in the central nervous system.

## Supplementary information

Supplementary information
