# Supplementary material for: The oncolytic adenovirus VCN-01 promotes anti-tumor effect in primitive neuroectodermal tumor models
Source: Sci Rep. 2019 Oct 7;9:14368. doi: 10.1038/s41598-019-51014-1 (PMC6779892; doi:10.1038/s41598-019-51014-1)
Supplement: Supplementary file 1 — Supplementary information [file 41598_2019_51014_MOESM1_ESM.pdf]

The oncolytic adenovirus VCN-01 promotes anti-tumor effect in primitive neuroectodermal tumor models

Marc García-Moure[1,2,3,] Naiara Martinez-Vélez[1,2,3,], Marisol Gonzalez-Huarriz[1,2,3,], Lucía Marrodán[1,2,3,], Manel Cascallo[4], Ramón Alemany[5], Ana Patiño-García[1,2,3,] and Marta M Alonso[1,2,3,]

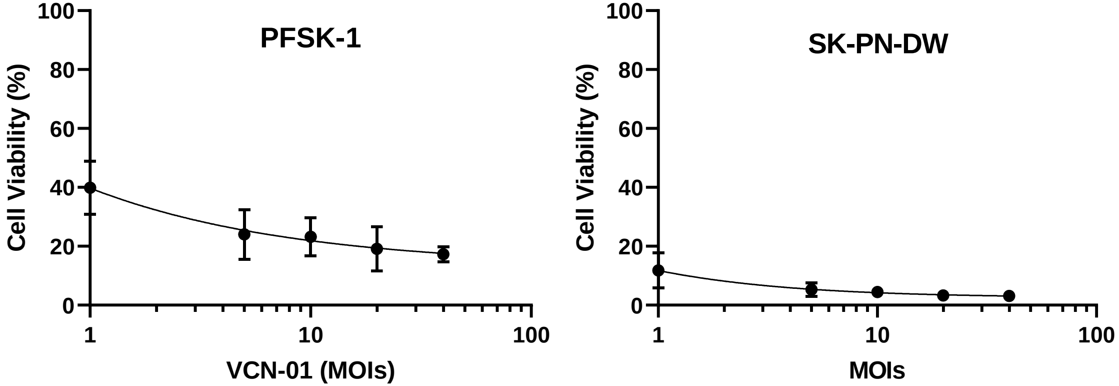

S1 Figure. VCN-01 treatment results in PNET cell death. Viability of PFSK-1 or SK-PN-DW cultures infected with VCN-01 at different MOIs ranging from 0 to 40 PFU/cell at 5 days post-infection, measured by propidium iodide staining. Graph indicates the percentage of viable cells relative to non-infected cultures, considered as 100% of viability (Mean ± SD; *n* = 3).
